# Structure reinvestigation of α-, β- and γ-In_2_S_3_

**DOI:** 10.1107/S2052520616007058

**Published:** 2016-05-26

**Authors:** Paul Pistor, Jose M. Merino Álvarez, Máximo León, Marco di Michiel, Susan Schorr, Reiner Klenk, Sebastian Lehmann

**Affiliations:** a Helmholtz-Zentrum Berlin, Germany; b Applied Physics Department, Universidad Autónoma de Madrid, Spain; c ESRF – The European Synchrotron, Grenoble, France; d Freie Universität Berlin, Berlin, Germany; e Solid State Physics, Lund University, Sweden

**Keywords:** indium sulfide, In_2_S_3_, tetragonal, cubic, trigonal, crystal structure analysis, lattice parameter, Rietveld refinement, thermal expansion coefficient, high temperature

## Abstract

Semiconducting indium sulfide (In_2_S_3_) has recently attracted considerable attention as a buffer material in the field of thin film photovoltaics. Compared with this growing interest, however, detailed characterizations of the crystal structure of this material are rather scarce and controversial. In order to close this gap, we have carried out a reinvestigation of the crystal structure of this material with an *in situ* X-ray diffraction study as a function of temperature using monochromatic synchrotron radiation. For the purpose of this study, high quality polycrystalline In_2_S_3_ material with nominally stoichiometric composition was synthesized at high temperatures. We found three modifications of In_2_S_3_ in the temperature range between 300 and 1300 K, with structural phase transitions at temperatures of 717 K and above 1049 K. By Rietveld refinement we extracted the crystal structure data and the temperature coefficients of the lattice constants for all three phases, including a high-temperature trigonal γ-In_2_S_3_ modification.

## Abstract 2

We report on the high-resolution structure analysis of In_2_S_3_ powder with monochromatic synchrotron light in the temperature range between 300 and 1300 K. Three modifications could be identified with the two phase transitions taking place at 717 K and above 1049 K. Crystal structure parameters and their temperature dependence for all three phases were extracted from the diffraction data by Rietveld refinement.

## 1. Introduction   

In_2_S_3_ is a widegap semiconductor with high photoconductive and photoluminescent properties, which makes it a promising material for optoelectronic applications (Shazly *et al.*, 1998 ▸). Most prominently, its potential application as a buffer layer in chalcopyrite solar cells has triggered an increased research effort in its fundamental materials properties (*e.g.* crystal structure, optical properties, electronic bandstructure *etc.*) as well as in deposition technology. The compatibility with various thin film deposition methods make it a versatile alternative to the commonly applied CdS buffer layer. Among the compatible deposition methods, atomic layer deposition (Naghavi *et al.*, 2003 ▸), the ion layer gas reaction (ILGAR) method (Sáez-Araoz *et al.*, 2012 ▸), spray pyrolisis (John *et al.*, 2005 ▸), sputtering (Hariskos *et al.*, 2005 ▸) and evaporation (Strohm *et al.*, 2005 ▸) have been successfully applied. The interested reader is referred here to the excellent review by Barreau (2009 ▸) on the role of In_2_S_3_  in the world of photovoltaics. Various reports on In_2_S_3_ buffer layers correlate deposition process parameters with crystallographic properties (Rao & Kumar, 2012 ▸; Larina *et al.*, 2004 ▸; Yoosuf & Jayaraj, 2005 ▸) and ultimately with final solar cell device parameters (Naghavi *et al.*, 2003 ▸; Pistor, Caballero *et al.*, 2009 ▸).

For a correct interpretation of the crystallographic data, a comprehensive understanding of the relevant crystal structure modifications of In_2_S_3_  is mandatory. Data reported on the phase labelling and temperature sequence in the In–S system is contradictory. In view of recent technological and scientific interest in In_2_S_3_ and the commonly drawn connection to its crystal structure properties, in this contribution we therefore report about a crystal structure reinvestigation of the In_2_S_3_ system in the temperature range from room temperature up to 1322 K, close to the melting point at 1363 K (Diehl *et al.*, 1976 ▸). The present study results in high quality reference powder diffraction data sets and enhanced knowledge on the different structure modifications of In_2_S_3_, which is expected to have a direct impact on the technological understanding in terms of *e.g.* material quality or diffusion parameters.

The first to describe the crystal structure of In_2_S_3_ were Hahn & Klingler (1949 ▸). They reported a cubic phase at temperatures below 600 K, which they called α-In_2_S_3_ and a transition to a tetragonal spinel-like high-temperature modification which they called β-In_2_S_3_. They already stressed the similarity between both modifications and suggested an ordering of the In atoms to be the main difference between the two phases. Later studies revealed that the cubic phase is in fact the higher-temperature phase and the stoichiometric phase existing at room temperature is tetragonal. However, for sulfur-deficient In_2_S_3_ the temperature range for the cubic phase extends down to room temperature. So Hahn probably measured a sulfur-deficient cubic α-In_2_S_3_ at room temperature and called it the lower temperature modification and from here most confusion about the labelling and temperature sequence of phases arises.

While the majority of authors follow Hahn in their nomenclature resulting in an ordering of the phases from low to high temperature as β–α–γ, some have relabeled the cubic and tetragonal phase with a resulting order of α–β–γ. Depending on the author, β-In_2_S_3_ in recent publications may therefore refer either to the low-temperature tetragonal phase or the cubic high-temperature phase. Although α–β–γ would be the logical order, we will follow the majority in the literature and assign β-In_2_S_3_ to the low-temperature, tetragonal phase.

There are some more and sometimes contradictory publications in the literature on the crystal structure of the different In_2_S_3_ phases, of which the most relevant ones will be shortly introduced in the following. Gödecke & Schubert (1985 ▸) suggest a phase diagram in which for the In:S ratio of 2:3 three modifications (β-In_2_S_3_, α-In_2_S_3_ and γ-In_2_S_3_) of indium sulfide exist in three different temperature regimes. King (1962 ▸) determined the space group of the tetragonal β-In_2_S_3_ as *I*4_1_/*amd* (space group No. 141) and the lattice parameters to 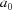 = 7.61 Å and 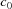 = 32.24 Å using Weissenberg photographs. The space group was later confirmed by Goodyear & Steigmann (1961 ▸) and Steigmann *et al.* (1965 ▸) who reported the lattice parameters as 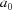 = 7.623 Å and 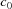 = 32.36 Å . Hahn described the space group of the cubic α-In_2_S_3_ as 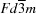, with a lattice parameter of 10.72 Å (Hahn & Klingler, 1949 ▸). The high-temperature γ-In_2_S_3_ modification has not been characterized in detail yet since a quenching to room temperature conditions was not successful (Diehl *et al.*, 1976 ▸). However, Diehl *et al.* succeeded in synthesizing a modified γ-In_2_S_3_ phase with an additional 5 at. % of As or Sb stabilizing it at room temperature. For this modified trigonal γ-In_2_S_3_ they suggested a space group of 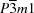 with lattice parameters 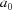 between 3.806 and 3.831 Å and 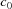 between 9.044 and 9.049 Å.

Some recent publications refer to the thin film application of In_2_S_3_ and cite X-ray diffraction (XRD) database information. An assignment to the tetragonal β-In_2_S_3_ is often made although the quality of the diffraction data for In_2_S_3_ is generally poor and does not allow for a definite distinction between the tetragonal β-In_2_S_3_ and the cubic α-In_2_S_3_. A correct distinction between the two phases would however allow for a verification of the film stoichiometry, as the β-In_2_S_3_ only exists in a very small stoichiometry range (Gödecke & Schubert, 1985 ▸). It is the scope of this work to reinvestigate the In_2_S_3_ in view of these aspects and to present a clear and detailed description of its crystal structure over the temperature range from room temperature to above 1300 K.

## 2. Experimental methods   

### 2.1. Sample preparation   

Indium sulfide was synthesized by heating weighted stoichiometric amounts of sulfur and indium in the ratio 3:2 in evacuated quartz ampoules. Source materials were indium granulate (> 99.999% purity) and sulfur flakes (> 99.999% purity). An excess of 4 atomic percent of S was provided for two reasons: (i) to account for sulfur losses during the preparation; (ii) the excess sulfur is not incorporated into the crystal structure according to the phase diagram (Gödecke & Schubert, 1985 ▸). The weighted source materials were filled in a graphite boat and placed in a quartz glass ampoule, evacuated (< 10^−3^ mbar) and sealed. The ampoule was placed in a two-zone oven and heated above the melting point to 1373 K for 24 h to enable the complete sulfurization of the indium. To assure that the synthesis was completed, the ampoule was then kept at 1073 K for 2 d, at 873 K for 2 d and at 673 K for 4 d. The synthesized indium sulfide was manually ground in a mortar before XRD measurements and had a brick red appearance.

### 2.2. Diffraction measurement   

For the diffraction measurement, ground indium sulfide powder was sealed in a quartz glass ampoule. The XRD measurements were performed at the ESRF, the European Synchrotron, Grenoble, France, at the beamline ID15B using monochromatic high-energy synchrotron light with a wavelength of 0.14276 Å. XRD patterns were recorded every 5 K, while heating the sample from 304 to 1322 K with a constant heating rate of 300 K h^−1^. The detector type used was a Pixium 4700, the detector-to-sample distance was 1037.132 mm, and the furnace was a resistively heated tube furnace.

### 2.3. Details of the Rietveld refinement   

The software *FullProf* Suite (February 2016 version) was used for the Rietveld refinement of the recorded diffractograms (Rodriguez-Carvajal, 2001 ▸). Apart from 7 profile parameters, the lattice parameters were fitted, as well as the atomic position coordinate parameters where appropriate, the isotropic temperature factor for all atomic positions, and the occupational factors of the indium positions. In addition we fitted the background by a list of manually inserted points which will add to the list of refined parameters.

## 3. Results   

### 3.1. Temperature-dependent phase analysis   

**Figure 1:**
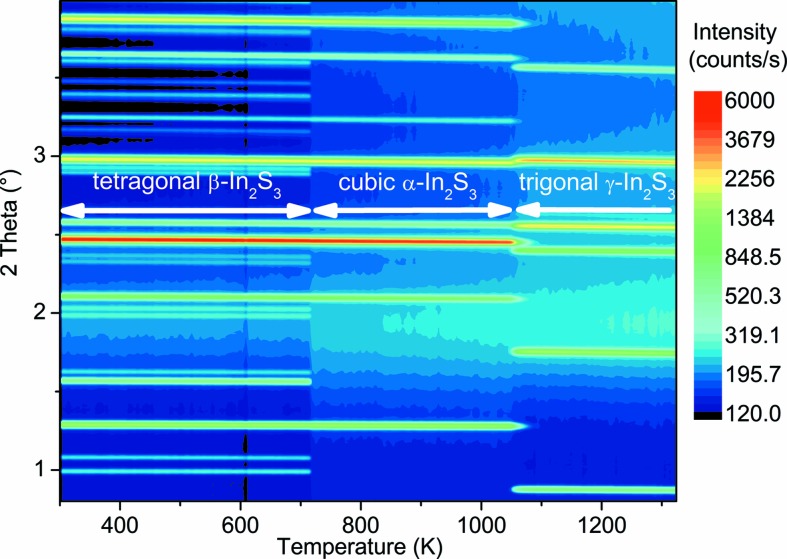
Map of temperature-dependent X-ray diffractograms (XRD) for the In_2_S_3_ powder. Each diffractogram is measured at a specific temperature which corresponds to one column in the graph with the *y*-direction displaying the 2θ diffraction angle. The position of the column in the *x*-direction corresponds to the temperature at which the diffractogram was recorded, while the colour indicates the XRD intensity in logarhythmic scale (dark blue = low intensity, red = high intensity). Three structural modifications of In_2_S_3_ in different temperature regimes can be distinguished according to appearing/disappearing diffraction peaks as indicated in the figure. The wavelength of the incident X-ray photons is 0.14276 Å.

In_2_S_3_ powder was prepared from the elements *via* a high-temperature route as described in §2.1. The mortared In_2_S_3_ powder was filled into quartz glass ampoules which were instantanously evacuated and sealed. The X-ray diffractograms were recorded in the temperature range from 304 to 1322 K, and details on the diffraction measurements can be found in §2.2. A colorscale map of all diffractograms is depicted in Fig. 1 ▸. In this graph, color indicates the counts and the three temperature ranges corresponding to the three different phases with distinct diffraction patterns can be well separated. We find a sharp structural phase transition between the first two phases at a temperature of 717 ± 5 K. This transition is characterized by the disappearance of several minor intensity diffraction peaks, while the main diffraction peak positions and intensities remain appoximately constant for both phases (see Figs. 1 ▸ and 2 ▸). A second transition was observed in the temperature range between 1049 and 1084 K. Here, all peaks of the α-In_2_S_3_ disappear and are replaced by the diffraction peaks of γ-In_2_S_3_ indicating a complete reordering of the atoms in the crystal structure.

### 3.2. Rietveld refinement of the three In_2_S_3_ modifications   

**Figure 2:**
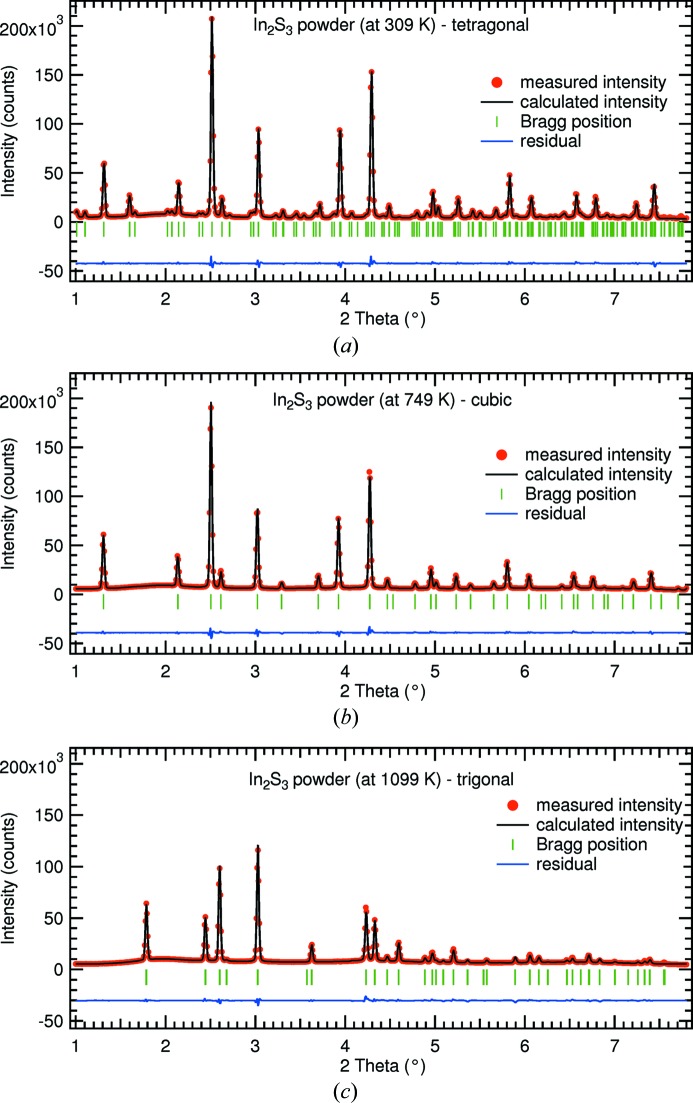
Diffraction data and Rietveld refinement of the three In_2_S_3_ modifications fully refined in this study. The displayed residua have been vertically shifted for better comparison. (*a*) Tetragonal modification measured at 309 K; (*b*) cubic modification measurement at 749 K; (*c*) trigonal modification measured at 1099 K.

We carried out full Rietveld refinements of the three diffractograms recorded at temperatures of 309, 749 and 1099 K. The refined lattice parameters are listed in Table 1 ▸. The three diffractograms, refinements and residues are displayed in Fig. 2 ▸. The calculated figures of merit and atomic positions can be found in Tables 2 ▸ and 3–5 ▸ ▸ ▸, respectively. For all three phases we obtained a good agreement between measurement and simulation with χ^2^ values below 11 and Bragg *R*_I_-factors below 0.03. More detailed information on the refinement parameters are included in the CIF file in the supporting information.

### 3.3. Temperature dependence of the lattice parameters   

**Figure 3:**
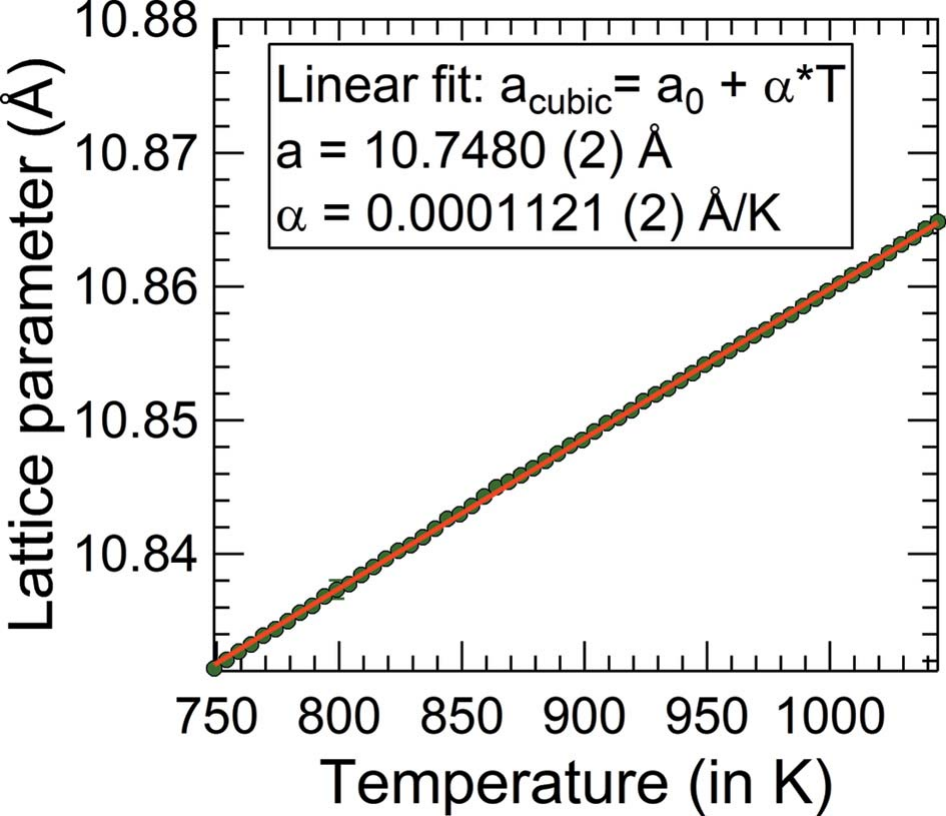
Temperature dependence of the lattice parameter for the cubic α-In_2_S_3_. The resulting fit parameters of a linear fit are listed in the inset.

Finally, the XRD data measured at different temperatures have been processed in batch-mode Rietveld refinements to extract the temperature dependence of the relevant lattice parameters. An example is shown in Fig. 3 ▸ for the lattice parameter *a*_0_ of the cubic α-In_2_S_3_ phase in the temperature range between 749 and 1044 K. The data are well fitted with the linear fit function
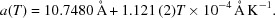
From the temperature dependence of the lattice constants, we obtain the linear thermal expansion coeffient α (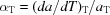; Kundra & Ali, 1976 ▸) for the cubic phase. In this temperature range, we calculate an average α of 10.3 × 10^−6^ K, in relatively good agreement with Kundra & Ali (1976 ▸), 10.8 × 10^−6^–10.9 × 10^−6^ K). The temperature dependence of the remaining lattice parameters of the tetragonal and trigonal phases are obtained accordingly and the fit functions are summarized in Table 6 ▸. For the tetragonal β-In_2_S_3_ we find an average linear expansion coefficient α in the *a* direction of 11.7 × 10^−6^ K and in the *c* direction of 6.7 × 10^−6^ K. For the trigonal phase, an average linear expansion coefficient of 14.1 × 10^−6^ K in the *a* direction and 26.7 × 10^−6^ K in the *c* direction was determined.

## 4. Discussion   

In this section we will briefly discuss the crystal structure of the three modifications and how the different modifications may impact the application of In_2_S_3_ in thin film solar cells.

**Figure 4:**
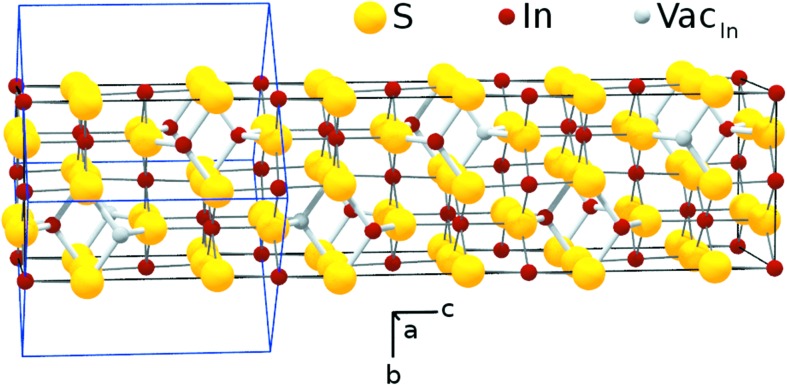
Structure model of a β-In_2_S_3_ unit cell. The tetrahedral bonds are drawn thicker for better identification. The indium vacancies are marked as grey spheres. In the tetragonal β-In_2_S_3_ configuration, the vacancies are ordered on a 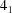 screw axis parallel to the *c*-axis of the crystal. In the α-In_2_S_3_ configuration, the vacancies are randomly distributed over all tetrahedral indium sites. The edges of the unit cell of the tetragonal β-In_2_S_3_ structure (cubic α-In_2_S_3_) structure are indicated by black (blue) lines.

The low-temperature modification β-In_2_S_3_ is best described with a defect spinel-type structure. The S atoms form a distorted cubic closed-spaced sublattice, in which the In atoms occupy the tetrahedral and octahedral interstitials the same way cations do in a regular spinel-like MgAl_2_O_4_ (Kleber *et al.*, 2002 ▸). While all the octahedral cation sites are occupied in β-In_2_S_3_, one third of the tetrahedal sites remain unoccupied. For that reason the In_2_S_3_ structure is sometimes described in a quasi-ternary compound formula: [In_2/3_(Vac)_1/3_]^tet^[In]
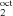
S_4_, where 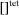 and 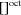 denote tetrahedral and octahedral sites and (Vac) the vacancies. In β-In_2_S_3_, the vacancies are ordered along the 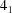 screw axis which is by definition parallel to the *c*-axis. The ordering of the vacancies gives rise to a small distortion of the cubic symmetry of the regular spinel structure. This small distortion is the origin of the tetragonal structure of the β-In_2_S_3_ with lower symmetry and leads to the additional peaks observed in the X-ray diffraction. Fig. 4 ▸ shows a plot of the β-In_2_S_3_ crystal structure based on the results obtained by the Rietveld refinement.

The transition from β-In_2_S_3_ to α-In_2_S_3_ is an order–disorder transition. In α-In_2_S_3_, the indium vacancies are randomly distributed over all tetrahedral sites, in contrast to the ordered configuration of vacancies in the β-In_2_S_3_. As a result of the disordering, α-In_2_S_3_ adopts a cubic crystal structure. The resulting higher crystal symmetry explains the observed disappearance of some of the minor intensity peaks in the diffractograms at the transition from β-In_2_S_3_ to α-In_2_S_3_ at 717 K.

Finally, the γ-In_2_S_3_ can be described as a layered structure as suggested by Diehl *et al.* and Bartzokas *et al.* (Diehl *et al.*, 1976 ▸; Bartzokas *et al.*, 1978 ▸). Here, the S atoms remain in a nearly closed-packed sublattice while the In atoms are exclusively found on octahedral sites forming a layered structure of subsequent S—In—S—In—S slabs.

The defect spinel-type structure of the β-In_2_S_3_ and α-In_2_S_3_ has a direct phenomenological and technological impact. Because of the large number of natural vacancies in the structure, In_2_S_3_ can host various other atoms such as Na or Cu within its original lattice configuration (Barreau *et al.*, 2006 ▸). Both have been found to diffuse efficiently through In_2_S_3_ thin films (Pistor, Allsop *et al.*, 2009 ▸; Juma, Pistor *et al.*, 2012 ▸; Juma, Kavalakkatt *et al.*, 2012 ▸). The diffusion phenomena at interfaces in thin film solar cells containing In_2_S_3_ have not yet been fully understood but might benefit from an in-depth knowledge of the crystal (vacancy) structure of In_2_S_3_. Where XRD data of good quality exist, it is an easy task to distinguish between the tetragonal and cubic phase of In_2_S_3_ by an examination of the characteristic additional peaks only present for the β-In_2_S_3_. The differentiation between α-In_2_S_3_ and β-In_2_S_3_ allows testing for stoichiometry, since the tetragonal phase only exists in a very small compositional range. According to Gödecke & Schubert (1985 ▸) and Diehl & Nitsche (1975 ▸), the compositional range for the β-In_2_S_3_ is less than 1 at %. The addition of a very small amount of surplus indium effectively surpresses the ordering of the In vacancies and therefore the formation of the tetragonal β-In_2_S_3_ phase. As a consequence, the crystal structure of off-stoichiometric 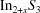 remains in the cubic α-In_2_S_3_ modification down to room temperature. This specific feature is used for example to check if In_2_S_3_ source material for an evaporation process is still within the described stoichiometry range (Pistor, Caballero *et al.*, 2009 ▸). Alike the distinction of polycrystalline powder material, this type of analysis tool would be rather desirable for the evalution of In_2_S_3_ thin film material as well. However, reasonable X-ray diffraction data are necessary to distinguish between the two very similar spectra in order to resolve the fine additional peaks, a criterion often not met for XRD patterns available on In_2_S_3_ thin films.

## 5. Conclusions   

We provide a detailed crystal structure analysis of In_2_S_3_ over the entire temperature range from room temperature up to close to the melting point covering the three modifications β-In_2_S_3_, α-In_2_S_3_ and γ-In_2_S_3_. With this, we contribute to the comprehensive understanding of the different phases existent and their interdependence. The high-temperature phase γ-In_2_S_3_ has been analysed and refined for the first time in the pure phase. Finally we show how the detailed knowledge of the phase diagram and the different In_2_S_3_ modifications might have a direct impact on the technological use of In_2_S_3_ in applications such as buffer layer deposition in thin film solar cell production.

## Supplementary Material

Crystal structure: contains datablock(s) global, TetragonalIn2S3, CubicIn2S3, TrigonalIn2S3. DOI: 10.1107/S2052520616007058/yb5011sup1.cif

Rietveld powder data: contains datablock(s) TetragonalIn2S3. DOI: 10.1107/S2052520616007058/yb5011TetragonalIn2S3sup2.rtv

Rietveld powder data: contains datablock(s) CubicIn2S3. DOI: 10.1107/S2052520616007058/yb5011CubicIn2S3sup3.rtv

Rietveld powder data: contains datablock(s) ppins_113_10160_counts, TrigonalIn2S3. DOI: 10.1107/S2052520616007058/yb5011TrigonalIn2S3sup4.rtv

CCDC references: 1476600, 1481751, 1481752

## Figures and Tables

**Table 1:** Lattice parameters as extracted from the Rietveld refinement for the three In_2_S_3_ modifications

		Temperature (K)	Space group	Number	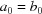 (Å)	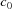 (Å)	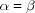 (°)	γ (°)
#1	β-In_2_S_3_	309	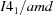	141	7.6231 (4)	32.358 (3)	90	90
#2	α-In_2_S_3_	749	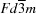	227	10.8315 (2)	10.8315 (2)	90	90
#3	γ-In_2_S_3_	1099	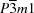	164	3.8656 (2)	9.1569 (5)	90	120

**Table 2:** Refinement parameters for the Rietveld refinements performed for the three modifications of In_2_S_3_ 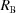
: Bragg 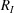 factor, 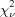: 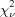 for points with Bragg contribution, 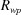: weighted profile *R*-factor (not corrected for background), 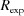: expected *R*-factor (not corrected for background), 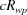: weighted profile *R* factor (corrected for background), 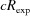: expected *R*-factor (corrected for background), 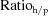: ratio between effective number of reflections and intensity parameters.

		Temperature (K)	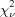	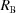	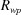	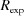	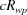	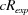	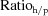
#1	β-In_2_S_3_	309	10.4	0.014	0.031	0.01	0.049	0.015	5.9
#2	α-In_2_S_3_	749	7.9	0.017	0.028	0.01	0.055	0.020	7.3
#3	γ-In_2_S_3_	1099	10.8	0.028	0.030	0.01	0.072	0.024	3.8

**Table 3:** Atomic sites for the tetragonal β-In_2_S_3_, (space group 141, origin choice No. 2)

Atom	Wickoff	*x*	*y*	*z*	*U*_iso_	Occ.
S1	16*h*	0	−0.005 (2)	0.2513 (7)	0.013 (4)	1.0
S2	16*h*	0	0.008 (2)	0.0777 (7)	0.016 (4)	1.0
S3	16*h*	0	0.020 (2)	0.4133 (7)	0.015 (4)	1.0
In1	8*e*	0	1/4	0.2046 (2)	0.0097 (8)	0.973 (6)
In2	8*c*	0	0	0	0.0143 (15)	0.972 (7)
In3	16*h*	0	−0.0196 (3)	0.3327 (2)	0.0111 (10)	0.974 (6)

**Table 4:** Atomic sites for the cubic α-In_2_S_3_ (space group 227, origin choice No. 2)

Atom	Wickoff	*x*	*y*	*z*	*U*_iso_	Occ.
S1	32*e*	0.2564 (2)	0.2564 (2)	0.2564 (2)	0.0347 (11)	1.0
In1	8*a*	1/8	1/8	1/8	0.0306 (9)	0.64 (4)
In2	16*d*	1/2	1/2	1/2	0.0445 (6)	0.978 (6)

**Table 5:** Atomic sites for the trigonal γ-In_2_S_3_

Atom	Wickoff	*x*	*y*	*z*	*U*_iso_	Occ.
S1	2*d*	1/3	2/3	0.3359 (7)	0.054 (4)	1.0
S2	1*a*	0	0	0	0.091 (5)	1.0
In1	2*d*	1/3	2/3	0.8085 (3)	0.0510 (9)	0.829 (10)
In2	2*d*	1/3	2/3	0.6485 (12)	0.064 (6)	0.144 (3)

**Table 6:** Linear fit function of the lattice parameters of In_2_S_3_ from the Rietveld refinements

		Temperature range (K)	Fit function
#1	β-In_2_S_3_	309–704	*a* = 7.5949 (2) Å + 8.967 (31)*T* × 10^−5^ Å K^−1^
			*c* = 32.307 (2) Å + 1.607 (4)*T* × 10^−4^ Å K^−1^
#2	α-In_2_S_3_	749–1044	*a* = 10.7480 (2) Å + 1.121 (2)*T* × 10^−4^ Å K^−1^
#3	γ-In_2_S_3_	1099–1322	*a* = 3.8044 (2) Å + 5.566 (15)*T* × 10^−5^ Å K^−1^
			*c* = 8.877 (3) Å + 2.52 (2)*T* × 10^−4^ Å K^−1^
